# Genome Sequences of *Arthrobacter* sp. Phages Inspire2, Ronnie, Hunnie, DeWayne, CGermain, Copper, Azathoth, and Arby

**DOI:** 10.1128/MRA.00575-19

**Published:** 2019-07-03

**Authors:** Melinda Harrison, Matthew D. Mastropaolo, Andrew Conboy, Cabrini University SEA-PHAGES Annotators, Lisa McKernan, Robert Schmidt, Véronique A. Delesalle

**Affiliations:** a Department of Science, Cabrini University, Radnor, Pennsylvania, USA; b Department of Mathematics and Sciences, Neumann University, Aston, Pennsylvania, USA; c Biological Sciences, Lehigh University, Bethlehem, Pennsylvania, USA; d Department of Biology, Chestnut Hill College, Philadelphia, Pennsylvania, USA; e Department of Biology, Gettysburg College, Gettysburg, Pennsylvania, USA; DOE Joint Genome Institute

## Abstract

Eight siphoviral phages isolated from various soil types and locations in southwestern Pennsylvania using *Arthrobacter* sp. strain ATCC 21022 were sequenced. The phages all have relatively small genomes, with each genome containing 15,556 bp. All 8 phages are closely related to previously described cluster AN *Arthrobacter* phages (K. K. Klyczek, J. A. Bonilla, D. Jacobs-Sera, T. L. Adair, et al., PLoS One 12:e0180517, 2017, https://doi.org/10.1371/journal.pone.0180517; J. Y. Lee-Soety, S. Bhatt, T. L. Adair, J. A. Bonilla, et al., Genome Announc 5:e01092-17, 2017, https://doi.org/10.1128/genomeA.01092-17).

## Abstract (precis)

Eight siphoviral phages isolated from various soil types and locations in southwestern Pennsylvania using *Arthrobacter* sp. strain ATCC 21022 were sequenced. The phages all have relatively small genomes, with each genome containing 15,556 bp. All 8 phages are closely related to previously described cluster AN *Arthrobacter* phages (K. K. Klyczek, J. A. Bonilla, D. Jacobs-Sera, T. L.

## ANNOUNCEMENT

*Arthrobacter* spp. are soil bacteria useful for their ability to protect themselves from heavy-metal toxicity and to survive long periods under stressful conditions induced by starvation, temperature shifts, ionizing radiation, oxygen radicals, and toxic chemicals (1–3). *Arthrobacter* phages contain diverse genomes, forming clusters from AK to FG and 68 singletons (4).

We have isolated and characterized an additional eight bacteriophages that infect *Arthrobacter* sp. strain ATCC 21022 (5). All the phages were isolated from soil samples by students in the Science Education Alliance-Phages Hunters Advancing Genomics and Evolutionary Science (SEA-PHAGES) program (6) at six different locations (Table 1) using an enrichment procedure (7). Soil samples were incubated with phage buffer, and bacteriophages were extracted from the mixture by filtering through a 0.22-μm filter. For virus replication, the filtered medium was incubated with *Arthrobacter* sp. ATCC 21022 at 30°C for 24 h. Plaque assays of isolated *Arthrobacter* phages resulted in small to medium plaques. Genomic DNA was isolated using a phenol-chloroform protocol (8).

**TABLE 1:** Cluster AN *Arthrobacter* sp. phages^*a*^

Phage name	GenBank accession no.	SRA accession no.	No. of reads	Location of isolation
Inspire2	MH576957	SRR9002785	1,000,000	Millstone, NJ
Ronnie	MH576961	SRR9002784	798,986	Langhorne, PA
Hunnie	MH576955	SRR9002786	42,889	Trevose, PA
DeWayne	MH576952	SRR9002787	1,600,000	Philadelphia, PA
CGermain	MH576950	SRR9002789	695,451	Philadelphia, PA
Copper	MH576951	SRR9002788	761,186	Peckville, PA
Azathoth	MH576948	SRR9002790	1,500,000	Philadelphia, PA
Arby	MK737943	SRR9002791	1,600,000	Chestnut Hill, PA

a Each phage has a genome length of 15,556 bp, a G+C content of 60.1%, 26 genes, and an 11-base 3' extension genome end type.

Sequencing, assembly, and finishing of the genomes were performed according to Russell (9). Phage genomes were prepared using the NEBNext Ultra II kit v3 and sequenced using the Illumina MiSeq platform using 150-bp unpaired reads. Sequences were assembled using Newbler 2.9 (10) with default settings, generating major contigs with coverages from 2,032- to 12,826-fold. Phage ends were determined as previously described (9) using Consed v29 (11) to check for completeness and accuracy of the termini. The genomes were annotated using DNA Master v5.23.3 (http://cobamide2.bio.pitt.edu/computer.htm), with coding sequences predicted by GeneMark v2.5p (12) and GLIMMER v3.02b (13), and 26 protein-coding genes were identified (Table 1). Phamerator (14) was used for comparative genomic analysis. No tRNA or transfer-messenger RNA (tmRNA) genes were detected by ARAGORN v1.2.38 (15) or tRNAscan-SE v2.0 (16). See Table 1 for genomic characteristics.

Each genome contains 26 putative genes. Given that the assignment of gene products to Phams was based on amino acid sequences similarity (4), all of the phages are closely related to each other with 100% gene content similarity, except for Arby, which shares 25 out of 26 Phams with our other 7 phages. Differences in the nucleotide sequences were also noted in the tape measure gene. All the genes in the genome are transcribed in the forward direction, except gene product 21, a helix-turn-helix (HTH) DNA-binding gene which is transcribed in the reserve direction (leftward). All genomes contain the typical structural and assembly genes, including the terminase, portal, capsid and protease fusion, tape measure, and major and minor tail proteins. Next, the lysis cassette contains 2 lysin genes, each with separate peptidase and amidase domains, followed by 4 genes encoding HTH DNA-binding motifs and an HNH endonuclease. The HTH gene GP23 N-terminal portion predicts strong coding potential and encodes the HTH domain (12). The GP23 C-terminal portion predicts very low coding potential with similarity to Vibrio cholerae O1 (12, 17). The poor-coding-potential region contains a series of inverted and direct repeats that are highly conserved, more than the HTH region, as well as an AT-rich region.

### Data availability.

GenBank accession numbers are provided in Table 1.
